# Disseminated intravascular coagulation in a dog naturally infected by *Leishmania* (*Leishmania*) *chagasi* from Rio de Janeiro – Brazil

**DOI:** 10.1186/1746-6148-9-43

**Published:** 2013-03-05

**Authors:** Carla O Honse, Fabiano B Figueiredo, Nayro X de Alencar, Maria de Fátima Madeira, Isabella DF Gremião, Tânia MP Schubach

**Affiliations:** 1 Laboratório de Pesquisa Clínica em Dermatozoonoses em Animais Domésticos, Instituto de Pesquisa Clínica Evandro Chagas, Fundação Oswaldo Cruz (Fiocruz), Av. Brasil, 4365, Manguinhos, Rio de Janeiro 21045-900, Brasil; 2 Laboratório Clínico Veterinário, Departamento de Patologia Clínica Veterinária, Faculdade de Veterinária, Universidade Federal Fluminense, Rua Vital Brasil Filho, 64, Niterói, 24230-340, Rio de Janeiro, Brasil; 3 Laboratório de Vigilância em Leishmaniose, Instituto de Pesquisa Clínica Evandro Chagas, Fundação Oswaldo Cruz (Fiocruz), Av. Brasil, 4365, Manguinhos, 21045-900, Rio de Janeiro, Brasil

**Keywords:** Disseminated intravascular coagulation, Dog, Visceral leishmaniasis

## Abstract

**Background:**

Disseminated intravascular coagulation (DIC) is an acquired disorder characterized by the activation of intravascular coagulation and excessive fibrin formation. It always occurs in association with other clinical conditions, including parasitic diseases. DIC has been described as a unusual complication in human and canine visceral leishmaniasis.

**Case presentation:**

DIC was found in a seven-year-old male mongrel dog naturally infected by *Leishmania* (*Leishmania*) *chagasi*. Haemostasis parameters demonstrated changes in primary and secondary haemostasis and fibrinolysis.

**Conclusion:**

DIC is a unusual condition described in canine visceral leishmaniasis and it seems to be associated with several immunological and pathological mechanisms involved in the disease.

## Background

Disseminated intravascular coagulation (DIC) is a syndrome characterized by the systemic activation of blood coagulation which generates intravascular fibrin leading to thrombosis in small and medium sized vessels and eventually organ dysfunction [1]. It can be also associated with severe hemorrhaging due to the consumption of platelets and coagulation factors [2]. DIC is not a primary disorder, always occurring in association with other clinical conditions, including sepsis, trauma, pregnancy complications, intravascular hemolysis, neoplasias, toxicity, hepatic diseases, cardiovascular diseases, parasitic diseases (protozoa) and others [3]. In human visceral leishmaniasis, DIC has been reported as an unusual complication [4]. In a review of hematological manifestations of visceral leishmaniasis, four human patients were associated with disseminated intravascular coagulopathy [5]. Blount et al. suggested that circulating immunocomplexes can directly activate the coagulation cascade in this disease [6].

The scarcity of publications regarding this disorder in canine visceral leishmaniasis [7,8] and the complex pathogenesis of both diseases, in which the liberation of inflammatory cytokines play important role in the response of host, motivated us to describe this clinical report of disseminated intravascular coagulation in a dog naturally infected by *Leishmania* (*Leishmania*) *chagasi* from Rio de Janeiro – Brazil.

## Case presentation

A seven-year-old male mongrel dog, weighing 10 kg, was taken to the Laboratory of Clinical Research on Dermatozoonosis in Domestic Animals, Evandro Chagas Clinical Research Institute – Oswaldo Cruz Foundation (Fiocruz), with a clinical suspicion of visceral leishmaniasis by Secretaria Municipal de Saúde do Rio de Janeiro (Municipal Health Department of Rio de Janeiro). The clinical examination revealed the presence of a single ulcerated cutaneous lesion with well-defined borders, measuring 0.5 cm in diameter, in the left ear, emaciation and lethargy. The mucous membranes were pale and the animal had signs of hepatomegaly and splenomegaly. There was no evidence of epistaxis, and the rectal temperature was 38.8°C.

Bleeding time (BT) was assessed with an automated spring-loaded device (2.4 mm - Unistik 2 Normal, Owem Mumford) in dry, clean and hairless skin of the inner right ear. Blood from the incision was blotted with Whatman nº2 filter paper every thirty seconds untill all bleeding stopped [9,10]. BT was 600 seconds (reference value 138 seconds).

Blood samples were collected by cephalic venipuncture and placed in three sets of tubes, one without anticoagulant for serological reassessment (Indirect ImmunoFluorescence – IIF), another with ethylenediaminetetraacetic acid (10 percent) for hematological evaluation and the third with sodium citrate (3.2 per cent) for coagulation tests. The serum sample was tested for anti-Leishmania antibodies with the IFI-Leishmaniose-Visceral-Canina-Bio-Manguinhos test (Bio-Manguinhos, Rio de Janeiro, Brazil) and the dog was seroreactive with titer ≥ 1/160. According to standards established by Ministério da Saúde (Health Department) [11], this canine patient was humanely euthanized with an intravenous administration of sodium thiopental 5%. After euthanasia, cutaneous lesion and spleen fragments were collected and immersed in saline containing 100 μg of 5’fluorocytocine, 1000 IU of penicillin and 200 μg of streptomycin per milliliter and stored at 4°C for 24 h. Afterwards, the fragments were transferred aseptically to a biphasic culture medium (NNN supplemented Schneider’s medium with 10% fetal bovine serum) and stored at 26–28°C. The fresh culture was monitored weekly for thirty days. Multi-locus enzyme electrophoresis (MLEE) was adopted for characterization of isolates, in accordance with Cupolillo et al. [12]. The parasite was isolated and, after isoenzyme analysis, was identified as *Leishmania* (*Leishmania*) *chagasi*.

The serum sample was also tested for the *Dirofilaria immitis* antigen as well as the antibodies against *Borrelia burgdorferi* and *Ehrlichia canis* (SNAP 3Dx – IDEXX), the results being negative.

Complete blood count (CBC) was performed with an automated cell counter (Coulter Model T-890) and the blood smears were stained by the Romanowsky method with Giemsa stain (Giemsa Stain Modified, Sigma-Aldrich). Hematological findings included PCV 26% (reference value 46%), RBC 3.6x10^6^/μL (reference value 6.5x10^6^/μL), hemoglobin 8.4 g/dL (reference value 15.4 g/dL), platelets 128x10^3^/μL (reference value 273×10^3^/μL) and total plasmatic protein 9.0 g/dL (reference value 7.1 g/dL).

Prothrombin time (PT) (Thromborel® S, Dade Behring) and activated partial thromboplastin time (APTT) (Pathromtin SL, Dade Behring) were determined with a fully-automated coagulation system – Sysmex Serie CA – 500 (Sysmex America, Inc.). A group of healthy dogs (n=18) was recruited for reference values of the hemostasis parameters. The animals, clinically and laboratory healthy (data not shown), were serologically negative for the *Dirofilaria immitis* antigen as well as antibodies against *Borrelia burgdorferi* and *Ehrlichia canis* (SNAP 3Dx – IDEXX) and not reactive to *Leishmania* sp. by the IIF test (Bio-Manguinhos, Rio de Janeiro, Brazil). PT and APTT were considered prolonged when the subject’s time was at least 30% longer than the healthy control’s time [2]. Hemostatic abnormalities included prolonged activated partial thromboplastin time 128.0 seconds (reference value, 37.2 seconds) and prolonged prothrombin time 9.8 seconds (reference value, 7.3 seconds).

The Dade® DIMERTEST latex assay was adopted for the rapid qualitative evaluation of circulating derivatives of fibrin degradation products (XL-FDP) (Dade Behring). The result was positive with sample agglutination.

## Discussion

Disseminated intravascular coagulation (DIC) is a hematological syndrome characterized by the activation of intravascular coagulation resulting in excessive fibrin formation and simultaneous consumption of coagulation factors and platelets resulting in severe hemorrhaging. DIC can be either acute (decompensated) or chronic (compensated) [13]. In general, several simultaneously occurring mechanisms play a role in the pathogenesis of disease. Fibrin deposition is a result of tissue factor/FVIIa complex-mediated thrombin generation and dysfunctional physiological anticoagulant pathways (mainly antithrombin III and protein C) [14]. A third important inhibitor of coagulation is tissue factor pathway inhibitor (TFPI), although its role in the pathogenesis of DIC is not clear. In addition, the inhibition of fibrinolytic activity by increased plasma levels of plasminogen activator inhibitor type 1 (PAI-1) results in a inadequate removal of fibrin contributing to thrombosis in small and medium sized vessels [14]. The activation and liberation of inflammatory cytokines in pathogenesis of DIC is unquestionable. According to van der Poll et al. [15], coagulation proteins (FXa, thrombin and Fibrin) can activate endothelial cells, stimulating the synthesis of proinflammatory cytokines and growth factor.

Several clinical conditions may lead to the development of DIC, including parasitic diseases. Activation of coagulation and subsequent fibrin deposition are essential parts of the host defense against infectious agents in an attempt to contain the invading microorganisms and the subsequent inflammatory response [1]. However, an exaggerated response can lead to a situation in which coagulation itself contributes to its most severe form of the disease causing microvascular thrombosis, organ dysfunction and bleeding [16]. Clinical signs such as epistaxis [17]–[19], haematuria [18,20] and disseminated intravascular coagulation [7] as well as laboratory abnormalities in haemostasis [7,8,21] have been reported in dogs with canine visceral leishmaniasis, suggesting that this protozoan affects not only primary and secondary haemostasis but fibrinolysis as well. In this report, the dog did not present clinical evidence of haemostatic abnormalities. This can be explained by a balance between destruction and production of coagulation factors and platelets resulting in a chronic or compensated status. The pathophysiology of compensated DIC and decompensated DIC is fundamentally the same, but acute DIC results from generation of a large amount of thrombin in a brief time period leading to hypercoagulable state and thrombosis, that can be followed by the development of a so-called hypocoagulable phase caused by depletion of clotting factors leading to bleeding, while the chronic DIC results of exposure of the coagulation system to small amounts of tissue factor, in which the coagulation factors and platelets are more able to be replaced in the majority of patients [13].

Hepatomegaly, splenomegaly and nephropathy have been often reported in dogs with canine visceral leishmaniasis [22,23]. *Leishmania* organisms can infect many different types of cells, including those of the mononuclear phagocityc system (macrophages and Kupffer cells), endothelial cells, hepatocytes, dendritic cells and others [24,25]. Activated mononuclear cells and endothelial cells induce expression of tissue factor that activates platelets and the coagulation system. Hepatomegaly is the result of inflammatory cell infiltration, permanent cell hypertrophy/hyperplasia and possible passive congestion [23,26]. Chronic granulomatous inflammation may be initially restricted within sinusoids and then expand to involve portal area, capsule or become diffuse [27,28], resulting in coagulation factor deficiency. Splenomegaly is caused by proliferation and/or infiltration of immune cells and is associated to hyperplasia of white and red pulp by changes in microvascular structure [29]. Spleen has been proposed as a key organ to allow long-term parasite survival due to an ineffective immune response and it has been correlated with the number of macrophages expressing complement receptors [30]. Nephropathy, which has been associated to immune-mediated mechanims, is characterized by presence of membranoproliferative glomerulonephritis and tubulointerstitial nephritis (rarely amyloidosis) [31] that can enable the loss of antithrombin III, a potent inhibitor of serine proteases in the coagulation cascate, contributing to the development of DIC. In the present case, clinical signs of hepatomegaly and splenomegaly were observed, but hepatic and renal enzymes and histopathological analysis were not performed.

As mentioned before, DIC is not a disease in itself, but always occurs secondary to an underlying disorder. Clinical evidence of metabolic disease, co-infeccions such as *Babesia* sp., neoplasias (e.g. lymphoma), immunological reactions, toxic reactions or trauma were not observed in the case reported here.

Previous studies on haemostasis in canine visceral leishmaniasis have obtained different results, indicating that many factors may play a role in the etiology of hemorrhagic manifestations, such as epistaxis, associated with the disease. According to Font et al. [7], epistaxis seems to be a result of inflammatory and ulcerative lesions of the nasal mucosa rather than changes in the clotting system. For Juttner et al. [32], other haemostatic defects may also contribute to epistaxis in dogs with leishmaniasis, particularly in the advanced stages of the disease. Petanides et al. [33] suggest that a single pathogenic mechanism can not be incriminated for epistaxis in natural canine visceral leishmaniasis, but rather the interaction of various internal factors.

Bleeding time is a less sensitive in vivo test of platelet function and its prolongation indicates platelet aggregation or adhesion defect. In this case, prolonged bleeding time can be attributed to vascular damage and thrombocytopenia. Domíngues and Torano [34] demonstrated that in the initial phases of infeccion, the *Leishmania* was able to interact directly with platelets by a specific mechanism causing formation of large aggregates. High concentrations of gammaglobulin may be associated with circulating immune complexes, common in canine visceral leishmaniasis [35,36], which may either be deposited on the vascular wall causing endothelial defects, platelet-platelet interaction disturbances and/or interaction deficiency between the platelets and the damaged subendothelium by coating the platelets and inhibiting degranulation and/or adhesion according to Juttner et al. [32]. Moreno et al. [17] reported an prolonged bleeding time in the presence of a normal platelet count in dogs with high plasma creatinine levels, attributing this finding to the platelet dysfunction associated with renal failure.

Thrombocytopenia has been related in canine visceral leishmaniasis [8,37,38] and ascribed to immune-mediated destruction triggered by the presence of immune complexes leading to the formation of anti-platelet antibodies, vasculitis resulting in a decrease of platelets [2] and the rapid consumption of platelets resulting from disseminated intravascular coagulation [7]. Unfortunately, it was not possible to evidence that these factors influenced thrombocytopenia developed by this dog, as well as hepatomegaly and splenomegaly. In other studies [17,32], a normal platelet count has been attributed in both stages of the disease (acute and chronic).

In the present study, both prolonged activated partial thromboplastin time and prothrombin time were apparent. Prolonged PT can be explained by disseminated intravascular coagulation with a reduction of the clotting factors involved in extrinsic and common pathways. Ciaramella et al. [19] observed normal PT values and attributed this finding to either the low sensitivity of the commercial PT tests on plasma canine or to a faster consumption of one or more of the intrinsic pathway clotting factors employed as activators in the formation of inflammatory chemical mediators [39]. Moreno [21] also declared normal m-OSPT (modified one-stage prothrombin time) possibly explained by the different degree of hepatic injury and the absence of disseminated intravascular coagulation. Badylak et al. [40] have reported prolonged one-stage prothrombin time in dogs with hepatic damage. In our case liver enzymes were not measured. Prolonged APTT can be justified by disseminated intravascular coagulation with a reduction in clotting factors involved in intrinsic and common pathways. This finding can also be related to renal failure or to liver disease, which may result in coagulation factor deficiency impairing the synthesis, although renal and liver enzymes were not measured in this case. Font et al. [7] reported a prolonged APPT in a dog with visceral leishmaniasis that had multiple hemorrhagic foci in the hepatic parenchyma. Moreno [21] also described prolonged APPT, even though bleeding was not always present. Valladares et al. [8] observed normal PT and APPT values in dogs with experimentally induced leishmaniasis. According to Ruiz de Gopegui et al. [41], PT and APTT within the reference interval may be seen appear in patients with early and compensated DIC.

The presence of circulating derivatives of fibrin degradation products can be linked to disseminated intravascular coagulation in this dog. In canine leishmaniasis, an increase in the concentration of fibrin degradation products has also been reported and accounted to either the renal loss of antithrombin III with a subsequent hypercoagulability state and increased fibrinolysis [42] or disseminated intravascular coagulation [7,8]. Moreno et al. [21] did not established differences in the levels of fibrin degradation products in their study.

## Conclusion

Several mechanisms can lead to activation of intravascular coagulation, including endotheial damage, platelet activation and release of tissue procoagulants. In canine visceral leishmaniasis, endotheial damage induced directly by Leishmania and the deposition of immuncomplexes appear to play a role in triggering disseminated intravascular coagulation. Thus, dogs naturally infected by *Leishmania* (*Leishmania*) *chagasi* can develop disseminated intravascular coagulation, which seems to be associated with several immunological and pathological mechanisms involved in the disease.

## Abbreviations

DIC: Disseminated intravascular coagulation; BT: Bleeding time; IIF: Indirect ImmunoFluorescence; NNN: Novy, MacNeal and Nicolle; MLEE: Multi-locus enzyme electrophoresis; CBC: Complete blood count; PCV: Packed cell volume; RBC: Red blood cell; FVIIa: Activated factor VII; TFPI: Tissue factor pathway inhibitor; PAI-1: Plasminogen activator inhibitor type 1; FXa: Activated factor X; PT: Prothrombin time; APTT: Activated partial thromboplastin time; m-OSPT: Modified one-stage prothrombim time

## Competing interests

None of the authors has any financial or personal relationships that could inappropriately influence or bias the content of the paper.

## Authors’ information

English review and revision by Mitchell Raymond Lishon, native of Chicago, Illinois, U.S.A. – U.C.L.A.1969.

## Authors’ contributions

COH and TMVP were responsible for the study design. COH and FBF were responsible for the collection of biological samples and clinical examination of the animal. NXA was responsible for the laboratorial parameters. MFM carried out the parasitological diagnosis and characterization of the parasite. COH and IDFG drafted the manuscript. COH, FBF, NXA and TMVP were involved in work supervision and writing of the manuscript. All authors read and approved the final manuscript. COH and TMVP are guarantors of the paper.
